# Increase of secondary mutations may be a drug-resistance mechanism for lung adenocarcinoma after radiation therapy combined with tyrosine kinase inhibitor

**DOI:** 10.7150/jca.35247

**Published:** 2019-08-29

**Authors:** Hongqing Zhuang, Siyu Shi, Yihang Guo, Zhongqiu Wang

**Affiliations:** 1 Department of Radiation Oncology, Peking University Third Hospital, Beijing, China; 2 Stanford University School of Medicine, Stanford, CA94305, US.; 3 Department of Radiotherapy, Tianjin Medical University Cancer Institute and Hospital, National Clinical Research Center for Cancer, Tianjin Key Laboratory of Cancer Prevention and Therapy, Tianjin, P.R. China

**Keywords:** erlotinib, radiation therapy, drug resistance, mutation

## Abstract

**Objective**: To investigate changes in the secondary mutations of tumor in a drug-resistance mechanism for lung adenocarcinoma after radiation therapy combined with tyrosine kinase inhibitor (TKI).

**Methods**: Lung adenocarcinoma cell line PC9 in vitro and xenograft model in nude mice were used to observe tumor inhibitory effects and drug-resistance under the effect of radiation therapy combined with erlotinib through apoptosis detection through in vitro survival curve and in vivo growth curve; changes in gene mutations before and after drug-resistance in nude mice xenografts were observed by the next generation sequencing, and the relationship between cancer drug-resistance and radiation therapy combined with TKI was observed.

**Results**: Radiation therapy combined with erlotinib had a more reliable radio-sensitizing effect in vitro and in vivo, however, there were several drug-resistant tumor cells. Meanwhile, radiation therapy combined with erlotinib could significantly increase the number of mutations in tumor genes. The whole genome sequencing showed that the secondary mutation in the combined treatment group significantly increased in comparison with those of the single treatment group and the blank control group.

**Conclusion**: The increase of secondary mutations may be an important drug-resistance mechanism for lung adenocarcinoma after radiation therapy combined with TKI, which provided further space exploration under the combined action of radiation and TKI.

## Background

Radiotherapy combined with tyrosine kinase inhibitor (TKI) has been extensively applied in clinical practice, and the combined action is common in drug-resistant patients 1-5. However, the majority of the current treatment strategies for such drug resistance refer to a simple TKI treatment, and there is a lack of research on the related mechanisms for drug resistance after the combined action 6-10. In this study, changes in the secondary mutations of tumor after radiation therapy combined with TKI were investigated, which resulted in providing new ideas for studies, associating with the mechanism of drug resistance in radiation combined with TKI, and it also presented reliable methods for solving such problems.

## 1. Materials and Methods

### 1.1 Cell line and nude mice

RPMI-1640 culture medium was purchased from Gibco (Billings, MT, USA), and fetal bovine serum was obtained from Sijiqing Biological Engineering Materials Co. Ltd. (Hangzhou, China). RNase A and propidium iodide (PI) were purchased from Sigma-Aldrich (St. Louis, MO, USA). The CO_2_ incubator used for cell culture was purchased from Heraeus (Germany, Frankfurt), in addition to the high-speed refrigerated centrifuge. The flow cytometer was purchased from Beckman Coulter Inc. (Brea, CA, USA). The PC9 lung adenocarcinoma cell line (exon EGFR19 mutation and KRAS wild-type) used in this study was obtained from Tianjin Medical University Cancer Institute and Hospital (Tianjin, China) 5-7. Cells were cultured in RPMI-1640 medium supplemented with 10% fetal bovine serum, 100 IU/ml penicillin, and 100 IU/ml streptomycin in an incubator at 37 °C with an atmosphere of 5% CO_2_. Cells in the exponential growth phase were irradiated as well 8-10.

### 1.2 Colony-forming analysis

Colony-forming rates of the tumor cells were determined by using the colony formation assay. The experiments on erlotinib-induced radiosensitization included the following treatment groups: blank control group, radiation alone group, erlotinib alone group, and combined erlotinib + radiation group. Cells in the exponential growth phase were trypsinized, counted, diluted, and seeded onto flasks (35 ml). The number of cells seeded onto the flasks was adjusted according to the radiation dose (500, 1000, 2000, 4000, 6000, 8000, and 10000 cells were seeded into 0, 1, 2, 4, 6, 8, and 10 Gy groups, respectively) 11-13. The concentration of erlotinib was 20 and 10 nM, respectively. After 14 days of cell seeding, the culture dishes were collected, and the culture medium was discarded. Cells were fixed and subjected to Giemsa staining. The number of colonies containing more than 50 cells was counted, and the cell survival fraction (SF) was calculated. The experiments were repeated by three times, and each treatment group contained three parallel samples 14. A single-hit-multi-target model was used to fit the cell survival curves.

### 1.3 Xenograft analysis

Here, PC9 cells were digested with exponential growth phase, in which counted and centrifuged at 1000 r/min for 5 min. Cells were then suspended, and about 1×10^6^ cells were injected into the thigh root of the nude mice. The tumors were observed 3 times per week after inoculation. When the tumor grew to about 1 cm, the experimental treatment was started. The groups were the same as the vitro experiment, and the radiation doses were the same to those in vitro. For in vivo experiment, both erlotinib and everolimus were used 13.

### 1.4 Apoptosis

Cell apoptosis was examined by flow cytometry. Experiments included the following treatment groups: blank control group, radiation alone group, erlotinib alone group, and combined erlotinib + radiation group. The concentration of erlotinib was the same as that listed in the preceding section. All irradiated groups were given a dose of 6 Gy. Colony-forming cells from different treatment groups were collected. Apoptosis was experimentally measured as follows: first, cells were trypsinized, and 5 × 10^5^ cells were collected. After adding 1 ml of cold phosphate buffered saline (PBS), the cells were centrifuged at 1000 rpm for 10 min at 4 °C. The cells were then washed with PBS, centrifuged twice under the above-mentioned conditions, and re-suspended in 200 μl of binding buffer. Also, 10 microliters of Annexin-FITC were added into the cell suspension and mixed well. The cell mixture was incubated at room temperature in the dark for 15 min, and then an additional 300 μl of binding buffer was added. Finally, the cells were analyzed by flow cytometry after adding 5μl of PI 14, 15.

### 1.5 Next generation sequencing

Large panel genome sequencing (more than 500 tumor-related genes): A customized Agilent's Sure Select Target Enrichment System was used to capture target regions with high coverage rate, which aimed to complete the target region sequencing of samples. A HiSeq PE150 sequencing was used, with an average of Q_30_>80%, and the average effective sequencing depth of samples was not less than 200X. Bioinformatics analysis of the generated sequencing data was performed by a software, and the whole genome sequencing was carried out as well; the DNA concentration was measured by Qubit DNA Assay Kit in Qubit® 2.0 Flurometer (Life Technologies, Carlsbad. CA, USA). A total amount of 1μg DNA per sample was required for sequencing library generation. The clustering of the index-coded samples was performed on a cBot Cluster Generation System by using a Hiseq X HD PE Cluster Kit (Illumina) according to the manufacturer's instructions. After cluster generation, the DNA libraries were sequenced on Illumina Hiseq X platform and 150-bppaired-end reads were generated. High-quality control was applied to guarantee the meaningful downstream analysis. These duplicate reads were uninformative and were not taken as evidence for variants into account. Picard was employed to mark these duplicates, so that GATK would ignore them in the following analysis 16,17.

### 1.6 Statistical analysis

Origin 7.5 software (Northampton, MA, USA) was used to fit the cell survival curves. Data were presented as the mean ± standard deviation (SD), and were analyzed by using SPSS 17.0 software (IBM, Armonk, NY, USA). The one-way analysis of variance (ANOVA) was used to make comparisons between multiple groups. P-value less than 0.05 was statistically considered significant.

## 2. Results

**Figure 1:**
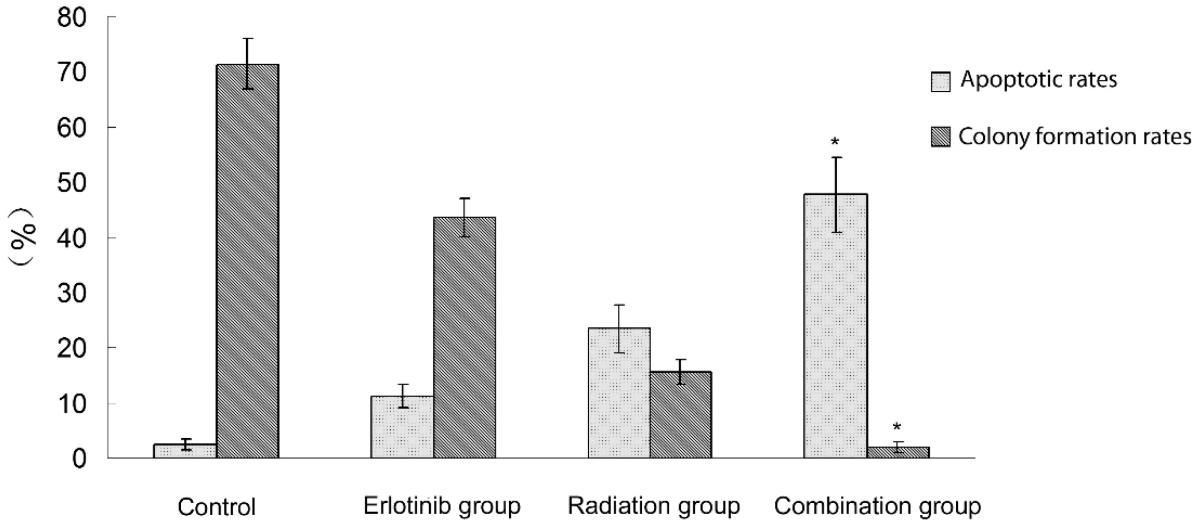
** Apoptosis and colony formation under the combined treatment of erlotinib and 6 Gy radiation.** Control group: the apoptotic rate was 2.43 ± 1.03%,and the colony formation rate was 71.45 ± 4.64%. Erlotinib group: the apoptotic rate was 11.26 ± 2.14%, and the colony formation rate was 43.56 ± 3.38%. Radiation group: the apoptotic rate was 23.45 ± 4.35%, and the colony formation rate was 15.6 ± 2.26%. In combined treatment of erlotinib and radiation group: the apoptotic rate was 47.68 ± 6.73%, and the colony formation rate was 2.04 ± 1.02%. There were statistical differences between the combination group and all other groups (* remarks).

**Figure 2:**
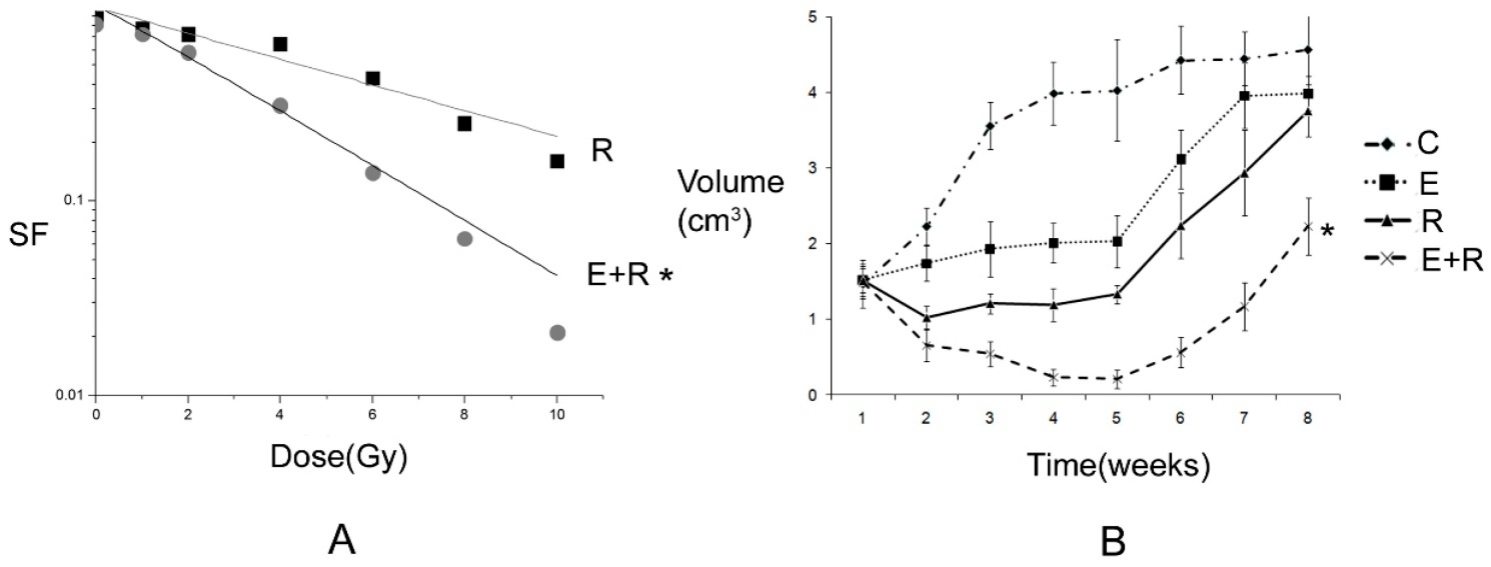
** Tumor inhibition and drug resistance after combination of erlotinib and radiation treatment.** A: The survival fraction of radiation alone combined with radiation and erlotinib groups in vitro. SER=2.18. P-value less than 0.05 was used for comparing two curves (* remarks). However, the final surviving fraction was 0.019±0.008, even after combination therapy. B: The growth curve of radiation alone combined radiation and erlotinib groups in vivo. When the curve of combination treatment compared with the other curves, all the P-values were less than 0.05 (* remarks). However, after 6 weeks, the volume of tumor was increased even under combination treatment. C: control group; E: erlotinib group; R: radiation group; E+R: combination erlotinib and radiation group.

2.1 Antitumor effect of radiation therapy combined with erlotinib and drug resistance The results of apoptosis detection showed that both erlotinib (11.26 ± 2.14%) and radiation (23.45 ± 4.35%) had inhibitory effects on tumor cells, and the apoptosis rate significantly increased after the combined action (47.68 ± 6.73%), suggesting that there was a synergistic effect between them. However, after incubation for a long-time, the survival curve of colony-forming analysis showed that in case of combination, the final survival fraction was about 0.019±0.01, and several drug-resistant tumor cells under the combined action and clones were formed (Figure 1). The results of xenograft test also showed that the growth curve under the combined action had a more reliable radio-sensitizing effect than that under the single treatment, while the volume of tumor slowly increased about 6 weeks after treatment (Figure 2). It can be concluded that radiation therapy combined with erlotinib had a synergistic effect on tumor inhibition; however, drug resistance may be the root cause of tumor progression in clinical practice.

### 2.2 Detection results of large panel gene

**Figure 3:**
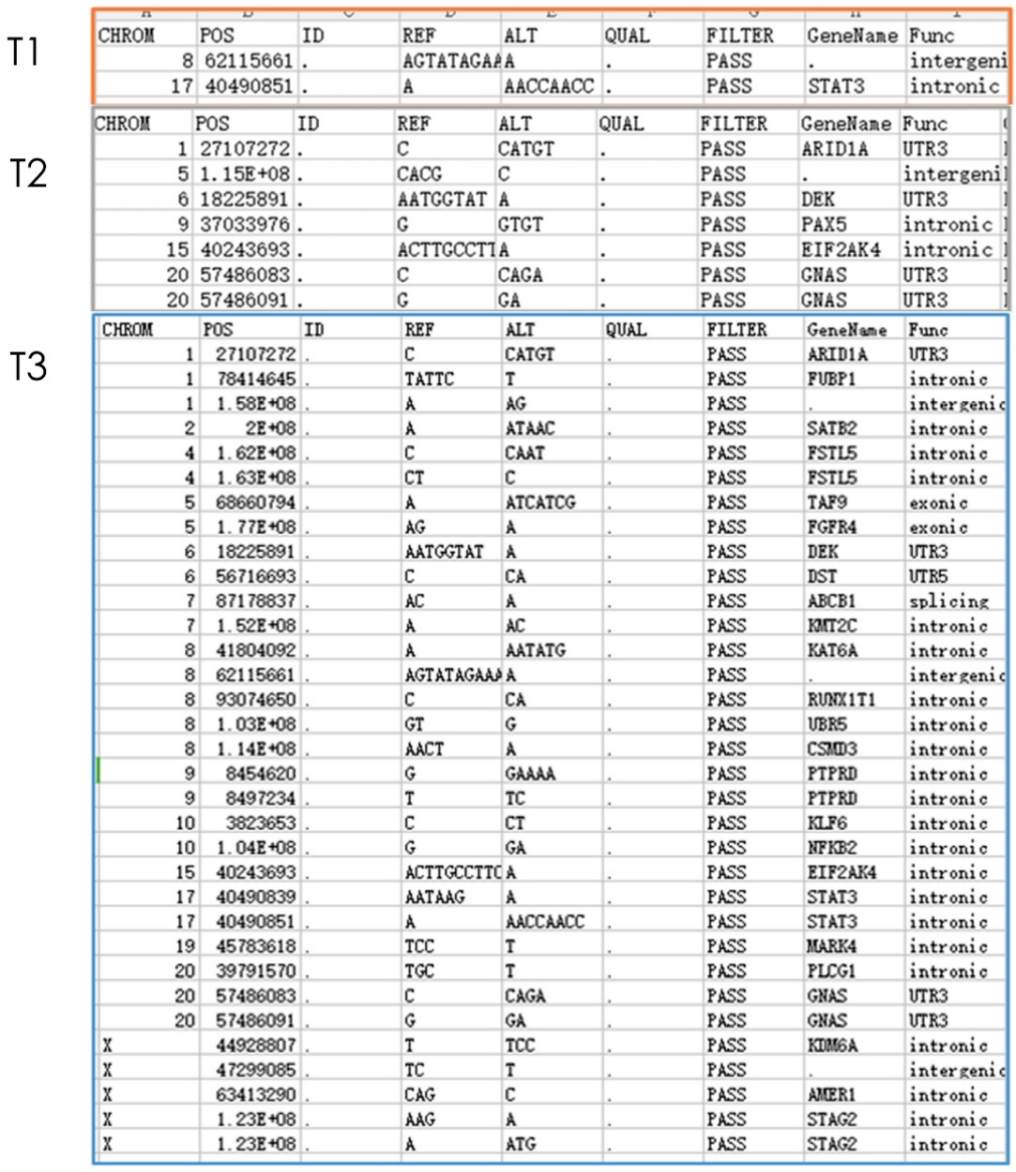
** Nest generation sequencing results of tumor-related genes:** T1: erlotinib alone group (2 mutations); T2: radiation alone group (7 mutations); T3: combined erlotinib + radiation group (31 mutations). It can be seen that after eliminating the original mutations in the blank control group, the number of cancer gene mutations in the combined treatment group was significantly higher than that in the single treatment group.

Gene detection of xenograft in nude mice showed that compared with untreated groups, the number of new mutation sites in the combined treatment group increased in comparison with erlotinib alone group and radiation alone group. Meanwhile, except for the original mutation sites that were the same as those in the blank control group, the mutation sites were different in each group. Hence, the radiation therapy combined with erlotinib may cause differences in gene mutation sites in the current detection of tumor-related genes (Figure 3).

### 2.3 Whole genome sequencing results

**Figure 4:**
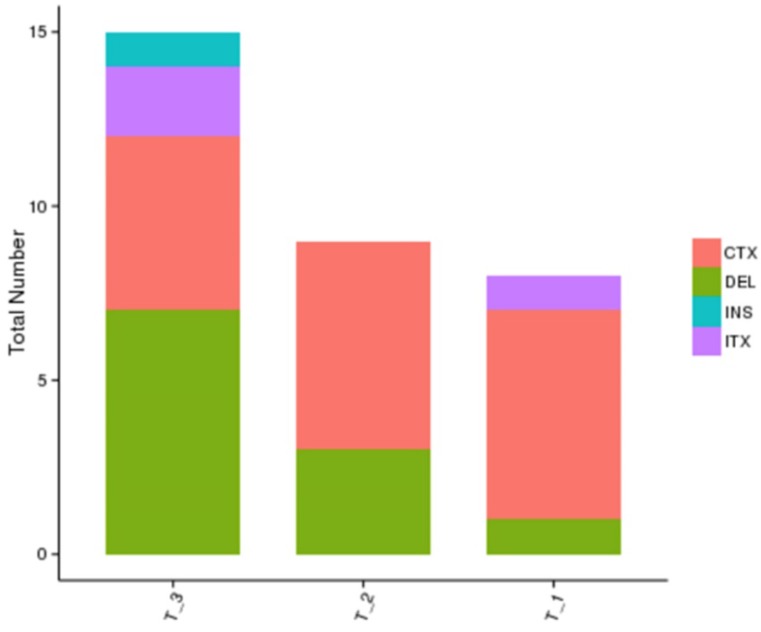
** Whole genome sequencing results of different treatment groups:** T1: erlotinib alone group; T2: radiation alone group; T3: combined erlotinib + radiation group. There were various mutation types (CTX: intrachromosomal translocation; DEL: delete; INS: insert; ITX: Interchromosomal Translocation) in the three different treatment groups, and there were similarities and differences in the mutation sites in each group as well. However, the number of cancer gene mutations in the combined treatment group was significantly higher than that in the single treatment group.

The whole genome sequencing results showed a more comprehensive image of new mutations in different treatment groups in comparison with the blank control group. The results showed that the number of gene mutations in the combined treatment group was more than that of the single treatment group, and the mutation sites were different after different treatments, which further showed the complexity of drug-resistance mechanism after radiation therapy combined with TKI (Figure 4).

## Discussion

The results of this study suggested that radiation therapy combined with TKI can induce more secondary mutations at gene loci, and the mechanism of drug-resistance was more complex, and that should be further studied.

The dual effects of radiation and TKI on tumor gene mutation are considered for secondary gene mutation after drug-resistance under the combined action. Radiation is an important factor, inducing gene mutation. Under radiation condition, secondary mutation of tumors can be significantly increased 17,18. At the same time, when TKI is used for a long-time, it also induces the related gene mutations. Moreover, the evolution of tumor heterogeneity and sub-clones can further differentiate cancer mutation. When radiation is combined with TKI, the secondary gene mutation of tumors may become more complex, demonstrating new challenges for the treatment of tumors 19-23.

Based on the current practice of cancer treatment, this study puts forward some issues for medical community to think about from the perspective of a basic research, which is of innovative significance 24,25. At present, the treatment of lung cancer in patients with TKI-resistant lung cancer who treated with or without radiotherapy refers to the simple TKI treatment. According to this study, the treatment considering the complexity of secondary drug-resistance mutation after radiation therapy combined with TKI may be biased. Therefore, further studies on the drug-resistance mechanism of radiation therapy combined with TKI need to be conducted 26.

Indeed, this is a preliminary study. The mechanism of drug-resistance under the combined action of radiation and TKI is still unclear, which needs to be further explored by basic and that should be further studied. Meanwhile, the results of a basic research need to be confirmed by clinical practice.

## Conclusion

In conclusion, the increase of secondary mutations may be an important drug-resistance mechanism for lung adenocarcinoma after radiation therapy combined with TKI. In this study, further space exploration for clinical practice under the combined action of radiation and TKI was provided, and we believe that with the help of an in-depth research and further accumulation of clinical data, we will have a deeper understanding of the drug-resistance mechanism of radiotherapy combined with TKI.

## Abbreviations

BED: Biological Effective Dose
NSCLC: Non-small-cell lung cancer
TKI: Tyrosine kinase inhibitors
